# Supplementary material for: Limosilactobacillus reuteri HCS02-001 Attenuates Hyperuricemia through Gut Microbiota-Dependent Regulation of Uric Acid Biosynthesis and Excretion
Source: Microorganisms. 2024 Mar 22;12(4):637. doi: 10.3390/microorganisms12040637 (PMC11052267; doi:10.3390/microorganisms12040637)
Supplement: Supplementary file 1 [file microorganisms-12-00637-s001.zip › microorganisms-2858433-supplementary.pdf]

Table S1. *In vitro* screening of gastrointestinal tolerance of candidate strains

Strain	strain number	Acid tolerance			Bile tolerance			Tolerance to artificial gastric juice			Tolerance to artificial intestinal juice		
		0h viable count（CFU/mL）	3h viable count（CFU/mL）	Survival rate（%）	0h viable count（CFU/mL）	3h viable count（CFU/mL）	Survival rate（%）	0h viable count（CFU/mL）	3h viable count（CFU/mL）	Survival rate（%）	0h viable count（CFU/mL）	4h viable count（CFU/mL）	Survival rate（%）
Lactobacillus rhamnosus	RH01103	（1.25±0.08）*10^{9}	(6.9±0.4)*10^{8}	55±1.47	（1.31±0.04）*10^{9}	（1.26±0.03）*10^{9}	96.15±0.73	（1.19±0.04）10^{9}	（1.18±0.04）*10^{9}	99.09±0.61	（1.32±0.03）*10^{9}	（1.31±0.02）*10^{9}	99.23±0.37
	RH01232	（9.33±0.07）*10^{8}	（3.49±0.15）*10^{8}	37.36±1.35	（1.07±0.03）*10^{9}	（9.3±0.3）*10^{8}	86.67±1.1	（9.81±0.04）*10^{8}	（9.51±0.03）*10^{8}	96.94±0.61	（9.88±0.04）*10^{8}	（8.42±0.11）*10^{8}	85.19±0.86
	RH01475	（1.18±0.03）*10^{9}	（5.4±0.08）*10^{8}	45.45±0.73	（1.09±0.03）*10^{9}	（1.01±0.03）*10^{9}	92.73±0.53	（1.24±0.03）*10^{9}	（9.9±0.2）*10^{8}	80±0.67	（1.18±0.03）*10^{9}	（1.09±0.02）*10^{9}	92.98±0.88
Limosilactobacillus reuteri	HCS02-001	（2.02±0.05）*10^{9}	（1.37±0.03）*10^{9}	67.65±0.98	（2.77±0.03）*10^{9}	（2.72±0.04）10^{9}	98.18±0.51	（2.29±0.03）*10^{9}	（2.22±0.01）*10^{9}	96.95±1.38	（2.23±0.02）*10^{9}	（2.19±0.01）*10^{9}	98.21±0.5
	RH02197	（1.29±0.11）*10^{9}	（6.8±0.6）*10^{8}	52.42±1.48	（1.11±0.03）*10^{9}	（9.6±0.2）*10^{8}	86.96±0.93	（1.2±0.02）*10^{9}	（1.05±0.03）*10^{9}	87.5±0.69	（1.35±0.04）*10^{9}	（1.27±0.03）*10^{9}	94.16±0.26
	RH02801	（2.37±0.04）*10^{9}	（1.51±0）*10^{9}	63.75±1.13	（2.43±0.04）*10^{9}	（2.18±0.01）*10^{9}	89.8±0.87	（2.35±0.03）*10^{9}	（1.87±0.03）*10^{9}	79.57±0.54	（2.46±0.03）*10^{9}	（2.14±0.04）*10^{9}	86.69±0.69
	RH02409	（2.48±0.04）*10^{9}	（1.49±0.01）*10^{9}	60±0.98	（2.82±0.04）*10^{9}	（2.72±0.06）*10^{9}	96.43±1.08	（2.72±0.02）*10^{9}	（1.96±0.03）*10^{9}	72.06±0.62	（2.62±0.04）*10^{9}	（2.27±0.03）*10^{9}	86.69±0.55
Lactiplantibacillus plantarum	RH03010	（1.92±0.03）*10^{9}	（1.03±0.03）*10^{9}	53.68±0.94	（2.8±0.02）*10^{9}	（2.65±0.04）*10^{9}	94.64±0.96	（1.9±0.02）*10^{9}	（1.76±0.01）*10^{9}	92.63±0.61	（2.34±0.03）*10^{9}	（2.34±0.04）*10^{9}	100±0.12
	RH03798	（1.86±0.03）*10^{9}	（0.99±0.01）*10^{9}	53.48±1.24	（1.93±0.02）*10^{9}	（1.48±0.02）*10^{9}	76.53±1	（2.12±0.04）*10^{9}	（1.15±0.03）*10^{9}	54.46±0.5	（2.27±0.05）*10^{9}	（2.2±0.06）*10^{9}	96.92±0.44
	RH03705	（1.25±0.05）*10^{9}	（5.7±0.1）*10^{8}	45.56±0.97	（1.12±0.03）*10^{9}	（1.05±0.02）*10^{9}	93.64±0.81	（1.23±0.02）*10^{9}	（1.05±0.02）*10^{9}	85.25±0.56	（1.32±0.05）*10^{9}	（1.21±0.05）*10^{9}	91.6±0.75
	RH03526	（2±0.02）*10^{9}	（7.4±0.2）*10^{8}	37.13±0.56	（2.3±0.02）*10^{9}	（1.5±0.03）*10^{9}	65.22±0.88	（2.54±0.03）*10^{9}	（1.84±0.02）*10^{9}	72.55±0.64	（2.64±0.03）*10^{9}	（2.33±0.03）*10^{9}	88.3±0.7
	RH03976	（1.35±0.03）*10^{9}	（5.6±0.1）*10^{8}	41.3±0.31	（1.5±0.03）*10^{9}	（1.15±0.04）*10^{9}	76.67±1.24	（1.5±0.02）*10^{9}	（（1.09±0.01））*10^{9}	72.67±0.81	（1.63±0.02）*10^{9}	（1.63±0.02）*10^{9}	81.48±0.4
Animal Bifidobacterium subsp. lactis	RH04020	（1.05±0.04）*10^{9}	（6.8±0.3）*10^{8}	65.09±0.55	（1.44±0.04）*10^{9}	（1.41±0.05）*10^{9}	97.93±0.87	（1.08±0.02）*10^{9}	（1.07±0.02）*10^{9}	99.07±0.67	（1.71±0.03）*10^{9}	（1.71±0.04）*10^{9}	100±0.75
	RH04568	（1.12±0.03）*10^{9}	（6.5±0.1）*10^{8}	57.79±0.83	（1.24±0.02）*10^{9}	（1.2±0.03）*10^{9}	96.75±0.66	（1.1±0.02）*10^{9}	（1±0.02）*10^{9}	90.9±1.37	（1.24±0.03）*10^{9}	（1.23±0.04）*10^{9}	99.2±0.69
Limosilactobacillus fermentum	RH08050	（1.81±0.03）*10^{9}	（9.8±0.2）*10^{8}	53.89±1.09	（2.04±0.06）*10^{9}	（2.02±0.05）*10^{9}	99±0.67	（1.99±0.04）*10^{9}	（1.95±0.05）*10^{9}	98±0.92	（1.86±0.04）*10^{9}	（1.72±0.04）*10^{9}	92.59±1.09
	RH08836	（1.5±0.02）*10^{9}	（5.3±0.1）*10^{8}	35.67±0.42	（1.42±0.02）*10^{9}	（1.03±0.01）*10^{9}	72.86±0.82	（1.75±0.03）*10^{9}	（1.56±0.05）*10^{9}	89.14±1.6	（1.64±0.03）*10^{9}	（1.5±0.04）*10^{9}	91.57±1.49
Lacticaseibacillus paracasei	HCS17-040	（1.26±0.03）*10^{9}	（9.6±0.1）*10^{8}	76.17±1.51	（1.3±0.02）*10^{9}	（1.26±0.03）*10^{9}	96.92±0.55	（1.27±0.03）*10^{9}	（1.2±0.03）*10^{9}	94.53±0.55	（1.22±0.04）*10^{9}	（1.22±0.04）*10^{9}	100±0.21
	RH17964	（1.62±0.02）*10^{9}	（8±0.2）*10^{8}	49.69±1.59	（1.8±0.02）*10^{9}	（1.45±0.03）*10^{9}	80.56±1.52	（1.98±0.03）*10^{9}	（1.85±0.04）*10^{9}	93.36±1.2	（1.9±0.02）*10^{9}	（1.7±0.03）*10^{9}	89.47±0.62
	RH17965	（1.41±0.02）*10^{9}	（9.3±0.1）*10^{8}	65.71±1.09	（1.45±0.02）*10^{9}	（1.3±0.02）*10^{9}	89.66±1.36	（1.2±0.03）*10^{9}	（1.01±0.02）*10^{9}	84.3±1.47	（1.5±0.04）*10^{9}	（1.39±0.05）*10^{9}	92.72±1.27
Pediococcus acidilactici	RH27102	（1.68±0.03）*10^{9}	（8.30±0.4）*10^{8}	49.4±1.35	（2.16±0.03）*10^{9}	（2.16±0.04）*10^{9}	100±1.09	（2.32±0.02）*10^{9}	（1.77±0.02）*10^{9}	76.17±0.96	（2.6±0.02）*10^{9}	（2.3±0.04）*10^{9}	88.46±1.14
	RH27865	（1.5±0.02）*10^{9}	（5.70±0.2）*10^{8}	38±1.37	（1.65±0.04）*10^{9}	（1.6±0.05）*10^{9}	96.97±1.08	（1.36±0.03）*10^{9}	（1.02±0.03）*10^{9}	74.81±0.82	（1.63±0.03）*10^{9}	（1.59±0.03）*10^{9}	97.53±0.82
Pediococcus pentosaceus	RH34011	（2.34±0.04）*10^{9}	（1.43±0.03）*10^{9}	60.87±0.96	（2.06±0.04）*10^{9}	（2.04±0.03）*10^{9}	99.02±0.87	（2.45±0.04）*10^{9}	（2.15±0.04）*10^{9}	87.76±1.29	（2.02±0.04）*10^{9}	（1.91±0.03）*10^{9}	94.5±1.29
	RH34985	（2.17±0.03）*10^{9}	（1.14±0.02）*10^{9}	52.31±0.66	（1.91±0.03）*10^{9}	（1.64±0.03）*10^{9}	85.86±0.78	（2.15±0.04）*10^{9}	（1.67±0.02）*10^{9}	77.46±1.21	（2.24±0.03）*10^{9}	（1.68±0.01）*10^{9}	75±0.96
